# DPSCs Protect Architectural Integrity and Alleviate Intervertebral Disc Degeneration by Regulating Nucleus Pulposus Immune Status

**DOI:** 10.1155/2022/7590337

**Published:** 2022-10-15

**Authors:** Xiwen Dong, Fanqi Hu, Jing Yi, Yuning Zhang, Chao Liu, Panpan Geng, Han Duan, Chu-Tse Wu, Xuesong Zhang, Hua Wang

**Affiliations:** ^1^Department of Experimental Hematology, Beijing Institute of Radiation Medicine, Beijing 100850, China; ^2^Medical School of Chinese PLA, Chinese PLA General Hospital, Beijing 100853, China; ^3^Department of Orthopaedics, the First Affiliated Hospital of Jinan University, Guangzhou 510630, China; ^4^Department of Orthopaedics, the Fourth Medical Centre, Chinese PLA General Hospital, Beijing 100853, China; ^5^Beijing Key Laboratory for Radiobiology, Beijing Institute of Radiation Medicine, Beijing 100850, China

## Abstract

Intervertebral disc (IVD) degeneration is the primary cause for low back pain that has a high prevalence in modern society and poses enormous economic burden on patients. Few effective therapeutic strategies are available for IVD degeneration treatment. To understand the biological effects of dental pulp stem cells (DPSCs) on nucleus pulposus (NP) cells, we carried out RNA sequencing, bioinformatic analysis which unveiled gene expression differences, and pathway variation in primarily isolated patients' NP cells after treatment with DPSCs supernatant. Western blot and immunofluorescence were used to verify these molecular alterations. Besides, to evaluate the therapeutic effect of DPSCs in IVD degeneration treatment, DPSCs were injected into a degeneration rat model in situ, with treatment outcome measured by micro-CT and histological analysis. RNA sequencing and in vitro experiments demonstrated that DPSCs supernatant could downregulate NP cells' inflammation-related NF-*κ*B and JAK-STAT pathways, reduce IL-6 production, increase collagen II expression, and mitigate apoptosis. In vivo results showed that DPSCs treatment protected the integrity of the disc structure, alleviated extracellular matrix degradation, and increased collagen fiber expression. In this study, we verified the therapeutic effect of DPSCs in an IVD degeneration rat model and elucidated the underlying molecular mechanism of DPSCs treatment, which provides a foundation for the application of DPSCs in IVD degeneration treatment.

## 1. Introduction

Low back pain (LBP) is a major public health concern, with a high prevalence in approximately 84% of individuals in their lifetime [1]. As the aging of the population has accelerated in recent decades, the socioeconomic costs caused by LBP are rapidly increasing. The total cost of LBP in the United States exceeds $100 billion per year [2]. Moreover, LBP is the second most frequent cause of hospital visits and the leading cause of disability in individuals younger than 45 years [3].

Degeneration of the intervertebral disc (IVD), soft tissue between the vertebrae that absorbs loads and lends flexibility to the spine, is recognized as a prime contributor (>40%) to LBP [4]. In the central part of the IVD is a hydrogel-like core, named the nucleus pulposus (NP). The NP can provide hydraulic pressure in all directions within each IVD. NP cells are the main type of cells residing in the NP and are responsible for synthesizing and producing a gelatinous extracellular matrix (ECM). The annulus fibrosus (AF) surrounds the NP and is a concentrically arranged lamella (composed of type I and type II collagen fibers) that keeps the NP in position during compression.

IVD degeneration is affected by various factors, including genetic factors, age, excessive manual labor, and infection. In the IVD degeneration process, increasing levels of inflammatory cytokines (such as TNF-*α*, IL-6, and IL-1*β*) upregulate genes encoding ECM-degrading enzymes, leading to increased aggrecan and collagen degradation [5]. The resulting inflammatory environment, NP cell depletion, and ECM degradation lead to a subsequent loss of hydrostatic pressure, causing structural changes and spinal instability and resulting in the subsequent leakage of NP material through annular fissures (disc herniation) and possibly spinal stenosis [6]. Disc herniation may cause infiltration of immune cells, neovascularization, and the appearance of nociceptive nerve fibers, which then lead to chronic pain. It is very difficult to reverse subsequent changes once the pathological process is initiated.

Amazingly, there are more than 200 treatments for LBP and IVD degeneration [7]. However, the average effects of conservative treatments for LBP are not much better than those of placebos [7]. Moreover, as a last resort, invasive surgical procedure (spine fusion or arthroplasty) is not an ultimate treatment option. The clinical success rates following spine fusion are generally reported to be between 50% and 70% [8]. An additional problem after successful fusion is the accelerated development of adjacent-level degeneration, which often requires additional surgery [9]. The limitations and defects of these treatments, notably the leading role of resident NP cell malfunction and depletion, warrant cell therapy as a new option for the treatment of IVD degeneration.

Dental pulp stem cells (DPSCs), an important component of mesenchymal stem cells (MSCs), may be suitable candidates for IVD degeneration treatment. Similar to MSCs, DPSCs have characteristics, such as plastic adherence under standard culture conditions; positive expression of cluster of differentiation (CD) 105, CD73, and CD90 surface molecules; negative/low expression of CD45, CD34, CD14 or CD11b, CD79 alpha or CD19, and human leukocyte antigen (HLA)-DR surface molecules; and the ability to differentiate into osteoblasts, adipocytes, and chondroblasts in vitro. Compared with other types of MSCs, the two major advantages of DPSCs are their high proliferative potential and potent immune-modulatory capacity [10–12] [13]. On the one hand, DPSCs possess high proliferative potential and can be passaged for more than 80 passages (the passage number of MSCs is usually less than 30) while maintaining their differentiation capacity [10, 14]; on the other hand, DPSCs can modulate the innate and adaptive immune systems by interacting with various components, such as T cells, natural killer (NK) cells, and macrophages [11]. Considering these characteristics, DPSCs may be a suitable candidate for IVD degeneration treatment.

In this study, human NP cells were isolated and treated with DPSCs' supernatant to understand the alteration of NP cells. Alterations in genes and pathways were elucidated by RNA-seq and western blot analysis. Besides, we used DPSCs to treat IVD degeneration rat model. Evaluations of the therapeutic effect of DPSC treatment were carried out radiologically and histologically to determine its effect on the IVD pathological structure and architecture. We discovered that DPSCs' supernatant could alleviate NP cell apoptosis, restore NP cell function, and reduce activation of the inflammatory pathway. In addition, DPSCs could improve the IVD structure and retard degeneration progression in an IVD degeneration rat model, all of which provide new insights as well as a novel strategy for IVD degeneration treatment.

## 2. Materials and Methods

### 2.1. Primary Human NP Cells Isolation

Human NP cells were obtained from surgical IVD tissue samples of patients (*n* = 6, male : female = 1 : 1, and average age = 30) enrolled in the People Liberation Army (PLA) General Hospital undergoing spinal surgery. All of the experimental protocols were in accordance with the Declaration of Helsinki and were approved by the Ethics Committee of the PLA General Hospital (S2019-113). Patients with spondylolisthesis, spinal stenosis, multiple disc herniation, or recurrent disc herniation were excluded. Routine MRI assessments of lumbar intervertebral disc degeneration were carried out by the Pfirrmann grading systems. Grade three disc degeneration was commonly observed in these patients. Primary human NP cells were isolated according to previously reported procedures [15]. In brief, patients' NP tissues were separated from the AF and washed with phosphate-balanced solution (PBS). To release NP cells, NP tissues were cut into pieces (2 mm^3^) and digested with collagenase II (0.25% w/v; Sigma, MO, USA) for 4 h at 37°C. The cell pellet was washed twice with PBS and resuspended in Dulbecco's modified Eagle medium (DMEM)/F12 (Gibco, NY, USA) containing 10% fatal bovine serum (FBS, Gemini, NY, USA) and 1% penicillin/streptomycin/amphotericin B. NP cells were then cultured at 37°C in a humidified atmosphere containing 5% CO_2_. After culturing for 10 days, NP cells were successfully isolated as passage 0. Cells at passages 2-3 were utilized in the following experiments.

### 2.2. Human Dental Pulp Stem Cells (DPSCs) Preparation

DPSCs were obtained from Beijing SH Biotechnology (http://www.bjshbio.com/) which were isolated and cultured as previously described [16, 17]. After washing with PBS, the cell pellet was resuspended and cultured in xenobiotic-free cell culture reagents. The characterization of DPSCs was performed as described in previous articles [16, 18]. DPSCs from passages 4-5 (approximately 20-25 divisions of primary DPSCs) were used in the experiments to achieve stable and reliable results.

### 2.3. Identification of DPSCs' Surface Marker

The phenotype of isolated DPSCs was evaluated by flow cytometry (FCM) for the expression of fluorescein isothiocyanate (FITC) Anti-Human CD45, FITC Anti-Human CD34, FITC Anti-Human CD19, phycoerythrin (PE) Anti-Human HLA-DR, PE Anti-Human CD73, and PE Anti-Human CD105 (Becton, Dickinson and Company, NY, USA).

### 2.4. Multipotential Differentiation

DPSCs' differentiation was induced in osteogenic or adipogenic medium (Cyagen Biosciences, CA, USA) according to manufacturing protocols. After induction, cells were fixed in 4% paraformaldehyde and stained with 1% Alizarin red for osteogenesis or oil red O for adipogenesis.

### 2.5. Western Blot Assay

NP cells in 6-well plates were washed with cold PBS three times and lysed in radioimmunoprecipitation assay (RIPA) lysis buffer (Beyotime, Shanghai, China) supplemented with premixed protease and phosphatase inhibitors (Beyotime, Shanghai, China) for 15 min on ice. The supernatant was collected by centrifugation at 13,000 g at 4°C for 20 min. Before electrophoresis, total proteins were quantified by the bicinchoninic acid protein assay (BCA, Thermo Scientific, Rockford, USA) and boiled for 8 min in loading buffer. Under constant voltage, proteins were resolved in 10% or 12.5% polyacrylamide gels before being transferred to polyvinylidene fluoride (PVDF) membranes. Then, the membranes were blocked in 3% bovine serum albumin (BSA) and incubated with the corresponding primary antibodies overnight at 4°C. After the membranes were washed in Tris-buffered saline containing Tween-20 (TBST) 4 times (5 min each), they were incubated with horseradish peroxidase (HRP) -conjugated secondary antibodies for 1 hr at RT. Signals were detected by the electrochemiluminescence (ECL) western blotting substrate (Solarbio Life Science, Beijing, China) and detected by a Tanon Imaging System (Tanon, Shanghai, Beijing). Each experiment was carried out at least twice.

The primary antibodies used in the immunoblot and immunoprecipitation assays were anti-phospho-inhibitor of Kappa B kinase (IKK, 1 : 1,000, Cell Signaling Technology, MA, USA), anti-phospho-signal transducer and activator of transcription 3 (STAT-3, 1 : 1,000, Cell Signaling Technology, MA, USA), anti-phospho-Akt (1 : 1,000, Cell Signaling Technology, MA, USA), anti-Akt (1 : 1,000, Cell Signaling Technology), anti-adenosine 5′-monophosphate (AMP)-activated protein kinase (AMPK, 1 : 1,000, Cell Signaling Technology), anti-STAT3 (1 : 1,000, Proteintech, Hubei, China), anti-cleaved-Caspase-3 (1 : 1,000, Proteintech), anti-Caspase-3 (1 : 1,000, Proteintech), anti-interleukin-6 (IL-6, 1 : 1,000, Proteintech), anticollagen II (1 : 1,000, Bioss, Beijing, China), antimatrix metalloproteinase 9 (MMP9, 1: 1,000, Bioss,), and antiglyceraldehyde-3-phosphate dehydrogenase (GAPDH, 1 : 1,000, Cell Signaling Technology).

The secondary antibodies used in the immunoblot assay were goat antirabbit IgG-HRP (1 : 6,000, Servicebio, Wuhan, China) and goat antimouse IgG-HRP (1 : 6,000, Servicebio).

### 2.6. Immunofluorescence Microscopy

NP cells were placed in confocal dishes (Nunc, Thermo Scientific, MA, USA) and treated for 48 hours. Cells were fixed with 4% formaldehyde for 30 min, permeabilized with 0.1% Triton X-100 for 20 min, and blocked with 3% BSA for 30 min. Then, the samples were incubated with rhodamine-phalloidin (Sigma-Aldrich, Oakville, ON, Canada) for 30 mins. Nuclei of NP cells were stained with 4,6-diamidino-2-phenylindole (DAPI) (Sigma-Aldrich, Oakville, ON, Canada). Fluorescence images were acquired with a Nikon N-STORM confocal microscope (Nikon Corporation, Tokyo Metropolis, Japan) using the NIS-Elements Viewer Software (Nikon Corporation, Tokyo Metropolis, Japan).

### 2.7. RNA Isolation, cDNA Library Preparation, and Sequencing

NP cells from patients were cultured in DMEM/F12 or DMEM/F12 containing DPSCs' culture supernatant (1 : 3 v/v) for 48 hours. Total RNA was subsequently extracted with TRIzol (Invitrogen, CA, USA) and chloroform-isopropanol and washed with 75% ethanol. Then, we used an Agilent 2100 Bioanalyser (Agilent Technologies, Santa Clara, CA, USA) and a Qubit Fluorometer (Invitrogen, CA, USA) to analyze the quality of the total extracted RNA. RNA samples were considered qualified for sequencing if they met the following standard: RNA integrity (RIN) >7.0 and a 28S:18S ratio>1.8. Capital-Bio Technology generated and sequenced the RNA-seq libraries (Beijing, China). Triplicate samples of all assays were constructed as independent libraries, and sequencing and analysis were performed. The NEBNext Ultra RNA Library Prep Kit for Illumina (NEB) was used to construct the libraries for sequencing. Poly(A)-tailed mRNA molecules were enriched by the NEB Next Poly(A) mRNA Magnetic Isolation Module (NEB) kit from 1 *μ*g total RNA. Then, mRNA was fragmented into ~200 base pair pieces. First-strand cDNA was synthesized from the mRNA fragments using reverse transcriptase and random hexamer primers, and then, second-strand cDNA was synthesized using DNA polymerase I and RNaseH. The end of the cDNA fragment was subjected to an end repair process that included the addition of a single “A” base, followed by ligation of the adapters. The products were purified and enriched by polymerase chain reaction (PCR) to amplify the library DNA. The final libraries were quantified using a KAPA Library Quantification kit (KAPA Biosystems, South Africa) and an Agilent 2100 Bioanalyser. After validation via quantitative reverse transcription-polymerase chain reaction (RT-qPCR), the libraries were subjected to paired-end sequencing with a paired-end reading length of 150 base pairs on an Illumina NovaSeq sequencer (Illumina).

### 2.8. Data Processing

Human genome version of hg38 was used as a reference. The quality of sequencing was checked using FastQC (v0.11.5), and then, low-quality data were filtered out by NGSQC Toolkit (v2.3.3) [19]. The clean reads were then aligned to the reference genome using HISAT2 (v2.1.0) with the default parameters. The processed reads from each sample were aligned against the reference genome by HISAT2. Gene expression analysis was performed with StringTie (v1.3.3b) [20]. DESeq (v1.28.0) [21] was used to analyze differentially expressed genes (DEGs) between samples. Thousands of independent statistical hypothesis tests were conducted on the DEGs separately. Then, a *p* value was obtained, which was corrected by the false discovery rate (FDR) method. The corrected *p* value (*q* value) was calculated by correction using the BH method. *p* values or *q* values were used to conduct significance analysis.

### 2.9. Bioinformatics Analysis

A heatmap was generated to illustrate significant alterations in gene expression between the control group and DPSCs' culture medium-treated group. We defined significantly altered genes as those genes with |log2FC| ≥1 and *p* value≤0.05. Functional classifications of the differentially expressed genes (DEGs) were performed with the Gene Ontology (GO) annotation (http://www.geneontology.org/), and the pathway analysis of the DEGs was performed with the Kyoto Encyclopedia of Genes and Genomes (KEGG), Reactome, or PANTHER databases. Based on the functional annotation from the GO database, the DEGs were assigned to the categories various biological process (BP), cellular component (CC), and molecular function (MF). Significant enrichment was defined as a *p* value<0.05. In addition, hierarchical cluster analysis was performed of the enriched genes by Cluster software.

### 2.10. Induction of the IVD Degeneration Rat Model

Sprague-Dawley rats (SD rats) were purchased from the Beijing Vital River Laboratory Animal Technology Co., Ltd. (Beijing, China). Animal experiments were approved by the Institutional Animal Care and Use Committee of Beijing Institute of Radiation Medicine (IACUC-DWZX-2020-718). Thirty-three 4-month-old male SD rats were housed in a SPF facility with a stable environment (temperature 22 ± 2°C, humidity 55 ± 10%, and 12-h light-dark cycle). Each cage contained three rats, which had free access to food and water at all times. Rats were randomly divided into three groups: normal (untreated) group, PBS treatment group, and DPSCs treatment group (*n* = 11 per group). After two weeks of accommodation, rat caudal intervertebral degeneration models were established using a previously described method [22]. Rats were anaesthetized with pentobarbital sodium before revealing the caudal intervertebral region, and all surgical procedures were performed under 2–3% inhaled isoflurane. Then, 20G needles were punctured into the Co7-8 or Co8-9 intervertebral disc through the center of the disc until the opposite side and were kept in place for 30 s. During surgery, one animal died from surgical complications. One rat was excluded due to tail falling off caused by severe infection. One week after the initial operation, 2 *μ*l of drugs (PBS and DPSCs) were injected into the intervertebral disc. The concentration of DPSCs was 5^∗^10^4^ cell/*μ*l. All experiments were performed under sterile conditions. The injection procedure was repeated after 4 weeks.

### 2.11. Microcomputed Tomography (Micro-CT) Imaging

Rat intervertebral discs were collected and analyzed by a high-resolution micro-CT imaging system (Quantum GX; PerkinElmer, MA, USA) to evaluate bone alterations. The parameters for micro-CT were set according to preliminary experiments, with a voltage of 70 kV and pixel size of 50.0 *μ*m. Three-dimensional reconstruction was carried out to demonstrate bone degeneration. Disc height was measured by Image-Pro Plus software (Media Cybernetics, MA, USA) and is expressed as the disc height index (DHI) using a previously described method [23, 24]. Briefly, DHI = (disc height at a depth of 25%of the vertebral bone width from the anterior + disc height at depth of 25%of the vertebral bone width from the posterior)/(vertebral bone height at a depth of 25%of the vertebral bone width from the anterior + vertebral bone height at a depth of 25%of the vertebral bone width from the posterior).

### 2.12. Histological and Immune-Histological Evaluation

Disc samples were harvested for histologic studies. Tissues were fixed in 10% neutral buffered formalin containing 10% cetylpyridinium chloride, decalcified in ethylenediaminetetraacetic acid (EDTA, Servicebio Technology, Wuhan, Hubei), paraffin-embedded, and sectioned (5 *μ*m) along the midsagittal plane. The sections were stained with hematoxylin and eosin for morphological evaluation, Masson's trichrome to determine the collagen fiber orientation, and safranin O-fast green to determine the proteoglycan distribution.

To evaluate histological changes, hematoxylin-eosin (HE) staining was scored by two pathologist blindly following previously reported criteria [25]. Briefly, the histological grading scale was based on 4 categories of degenerative changes (concerning the annulus fibrosus, the border between the annulus fibrosus and nucleus pulposus, cellularity of the nucleus pulposus, and matrix of the nucleus pulposus) with scores ranging from a normal disc with 4 points (1 point/category) to a severely degenerated disc with 12 points (3 points/category).

In addition, the architectural parameters of the IVD (such as intervertebral disc height (DH), maximum nucleus pulposus height (NPH), superior endplate heights (SEPH), and inferior endplate heights (IEPH)) were measured as previously reported by two pathologist blindly [26]. After obtaining the parameters, we calculated different ratios, such as the NPH/DH index, SEPH/DH index, IEPH/DH index, SEPH/IEPH index, and (SEPH+IEPH)/DH index.

Immunohistochemical (IHC) staining was carried out using a streptavidin-peroxidase kit (Zymed, CA, USA) in accordance with the manufacturer's protocols. Samples were blocked with 5% (w/v) BSA, incubated with primary antibodies against collagen II (Proteintech, Wuhan, China) overnight, and incubated with the corresponding secondary antibodies conjugated with biotin and diaminobenzidine (Beyotime, Shanghai, China). Finally, hematoxylin was used to stain nuclei.

### 2.13. Statistical Analysis

Statistical analysis was carried out by SPSS. Multiple group comparisons were performed by one-way analysis of variance (ANOVA) followed by Bonferroni's multiple comparison test (for data that had a Gaussian distribution) or by the Kruskal–Wallis test followed by Dunn's multiple comparison test (for data that did not have a Gaussian distribution). Graphs were made by GraphPad Prism 7. In the graphs, data are expressed as the mean ± SD. *p* values <0.05 were considered statistically significant.

## 3. Results and Discussion

### 3.1. Overview of RNA-Seq Data

**Figure 1:**
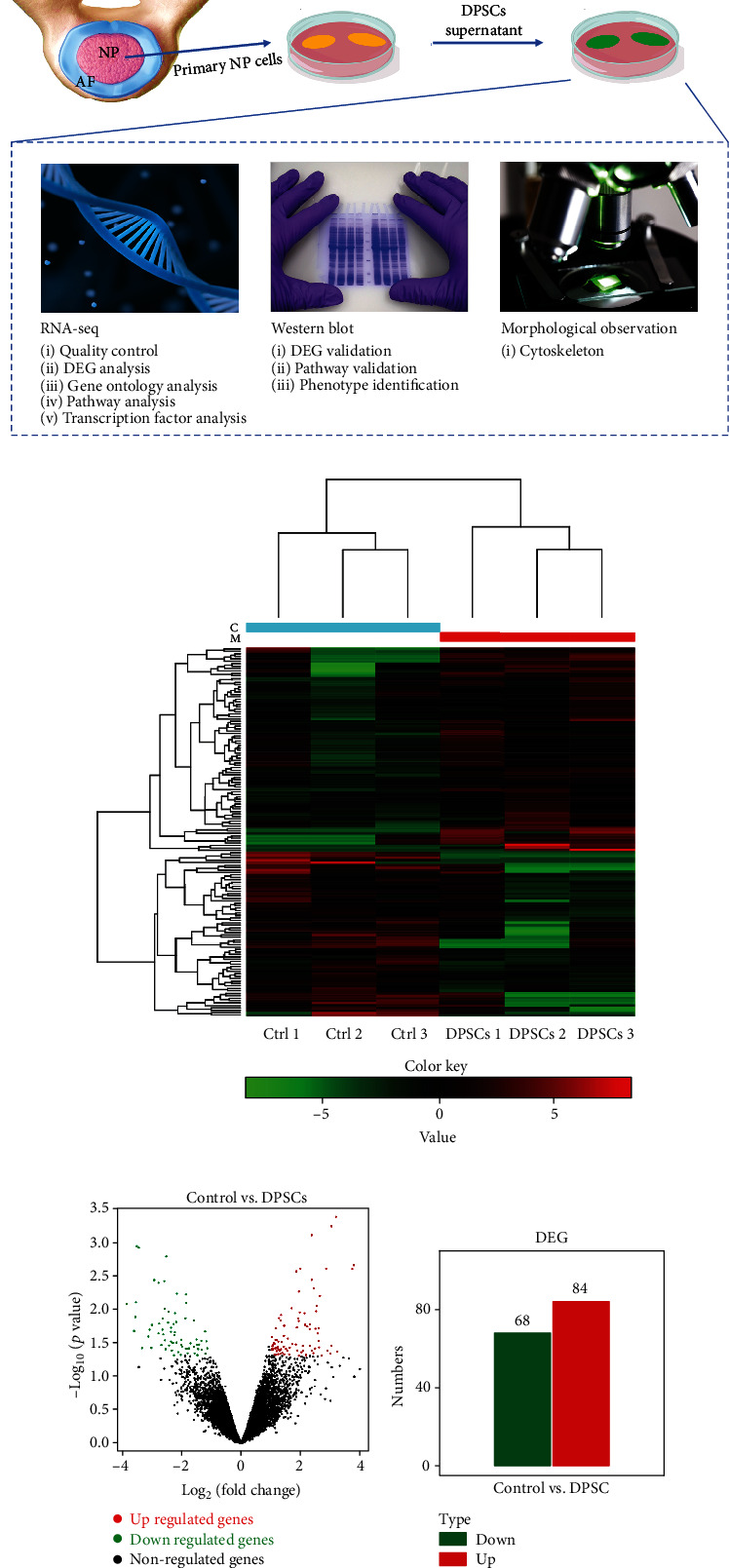
RNA-seq and detection of NP cell alterations after DPSC treatment. (a). Schematic map showing the study design to determine the underlying molecular mechanism of the DPSC treatment. Primary NP cells were isolated from IVD degeneration patients and treated with DPSC supernatant. Then, RNA-seq was carried out to determine the DEGs and pathways that were altered after treatment. Then, western blotting and confocal microscopy imaging were carried out to verify the cellular alterations. (b) Heatmap of the DEGs identified by RNA-seq after DPSC treatment. High and low abundances of gene expression are shown in red and green, respectively. (c) Volcanic product distribution map of the DEGs in NP cells. DEGs are marked with red (upregulated) or green (downregulated) dots. (d) Bar graph showing the number of DEGs. RNA-seq: RNA sequencing; DEGs: differentially expressed genes.

The identification of DPSCs' markers and differentiation ability are shown in Supplementary Figure 1. To better understand the alteration of NP cells after DPSCs' treatment, we detected changes in gene expression, signal pathways, and cell morphology following the schematic diagram shown in Figure 1(a). Briefly, primary NP cells were isolated from IVD degeneration patients and were then treated with or without DPSCs' supernatant. RNA sequencing (RNA-seq) and western blotting were carried out to determine the altered genes and pathways in the different groups, while immunofluorescence was carried out to detect morphological changes (Figure 1(a)).

NP cells were sequenced by an Illumina NovaSeq sequencer. After quality trimming by FastQC, clean data were acquired. Since the primer/adaptor contaminated reads, low-quality reads, and N overtop reads were filtered out, the clean reads had better quality than the individual forward or reverse reads (Supplementary Table 1). With respect to alignment, the average percentage of paired reads was 97.87% (all data >70%), and the average percentage of multiple paired reads was 3.21% (all data <10%), which met the quality criteria for subsequent analysis (Supplementary Table 2).

### 3.2. DEG Analysis Reveals Differential Gene Expression after DPSC Supernatant Treatment

We next determined the DEGs in DPSCs' supernatant-treated NP cells. DEG screening detected 68 genes that were significantly downregulated and 84 genes that were significantly upregulated. The expression heatmap of the DEGs is shown in Figure 1(b). A volcano plot and bar graph were used to display the distribution of the DEGs, as shown in Figures 1(c) and 1(d), respectively.

### 3.3. GO Function Analysis of DEGs

**Figure 2:**
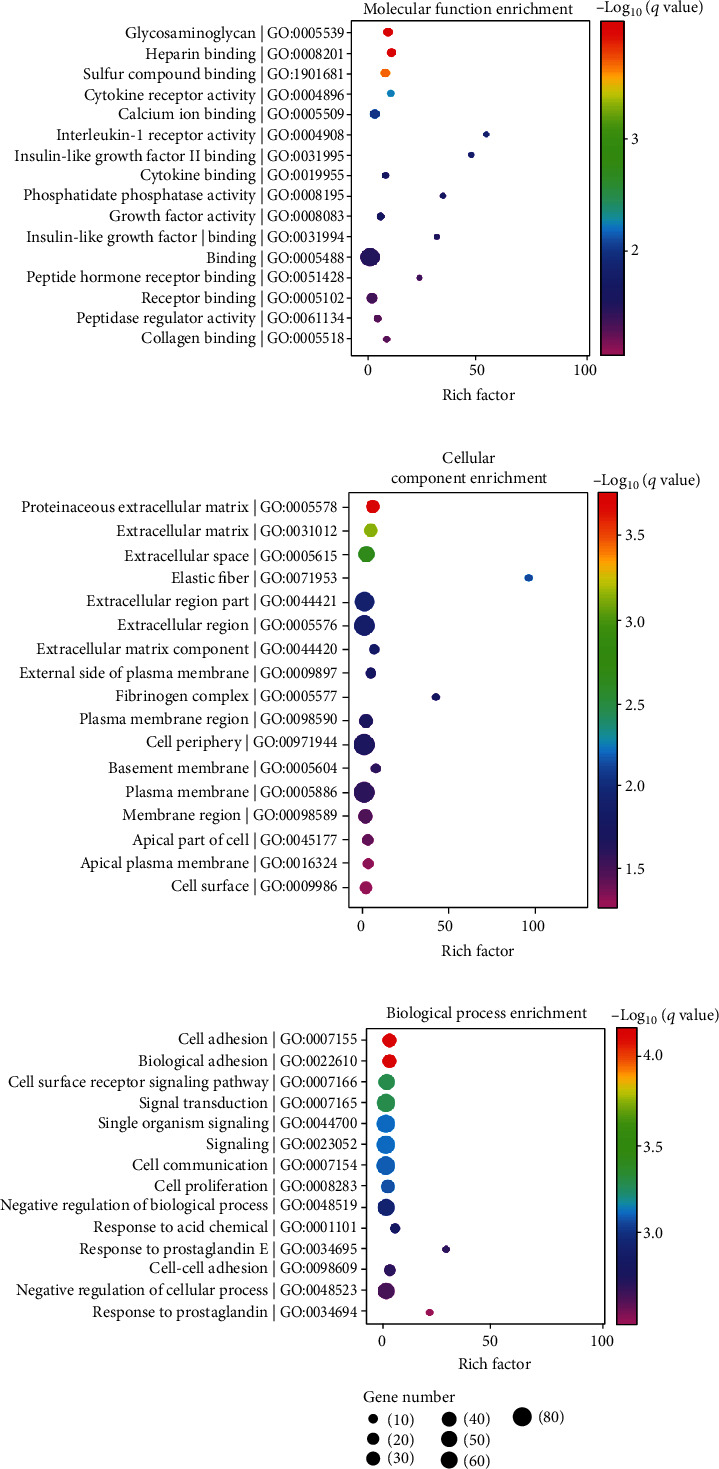
GO annotation of the DEGs from control NP cells and NP cells after DPSC supernatant treatment. (a) GO annotation of the DEGs most enriched terms in “molecular function.” (b) GO annotation of the DEGs most enriched terms in “cellular component.” (c). GO annotation of the DEGs most enriched terms in “biological process.” DEGs: differentially expressed genes; GO: gene ontology.

GO classification and functional enrichment analysis were performed to determine the functions of the DEGs and to characterize their functional distribution in the DPSCs' supernatant-treated group. The GO function classification results for the DEGs are shown in Supplementary Figure 2A. The GO functional enrichment results (concerning molecular function, cellular component, and biological process) for the DEGs are shown in Figures 2(a)–2(c).

For molecular function enrichment (Figure 2(a)), the top two terms with the highest richness factor were “interleukin-1 receptor activity” (2/7, input genes/background genes, corrected *p* value =0.014643) and “insulin-like growth factor II binding” (2/8, input genes/background genes, corrected *p* value =0.016888); the top two terms with the highest statistical significance were “glycosaminoglycan binding” (10/202, input genes/background genes, corrected *p* value =0.000106) and “heparin binding” (9/156, input genes/background genes, corrected *p* value =0.000106).

For cellular component enrichment (Figure 2(b)), the top two terms with the highest richness factor were “elastic fiber” (2/4, input genes/background genes, corrected *p* value =0.007240) and “fibrinogen complex” (2/9, input genes/background genes, corrected *p* value =0.018548); the top two terms with the highest statistical significance were “proteinaceous extracellular matrix” (12/352, input genes/background genes, corrected *p* value =0.000201) and “extracellular matrix” (12/426, input genes/background genes, corrected *p* value =0.000751).

For biological process enrichment (Figure 2(c)), the top two terms with the highest richness factor were “response to prostaglandin E” (4/26, input genes/background genes, corrected *p* value =0.001879) and “response to prostaglandin” (4/35, input genes/background genes, corrected *p* value =0.003010); the top two terms with the highest statistical significance were “cell adhesion” (25/1412, input genes/background genes, corrected *p* value =0.000074) and “biological adhesion” (25/1419, input genes/background genes, corrected *p* value =0.000074).

The GO hierarchy for molecular function is presented in Supplementary Figure 2B. “Molecular transducer activity” (*p* = 0.068324) and “binding” (*p* = 0.001988) were the two most significantly changed superior terms. For the term “molecular transducer activity,” “interleukin-1 receptor activity” (*p* = 0.014643) was the significantly enriched inferior term. For the term “binding,” “heparin binding” (*p* = 0.000106), “growth factor activity” (*p* = 0.023981), and “insulin-like growth factor II binding” (*p* = 0.016888) were the significantly enriched inferior terms.

### 3.4. Pathway Alteration after DPSCs' Supernatant Treatment

**Figure 3:**
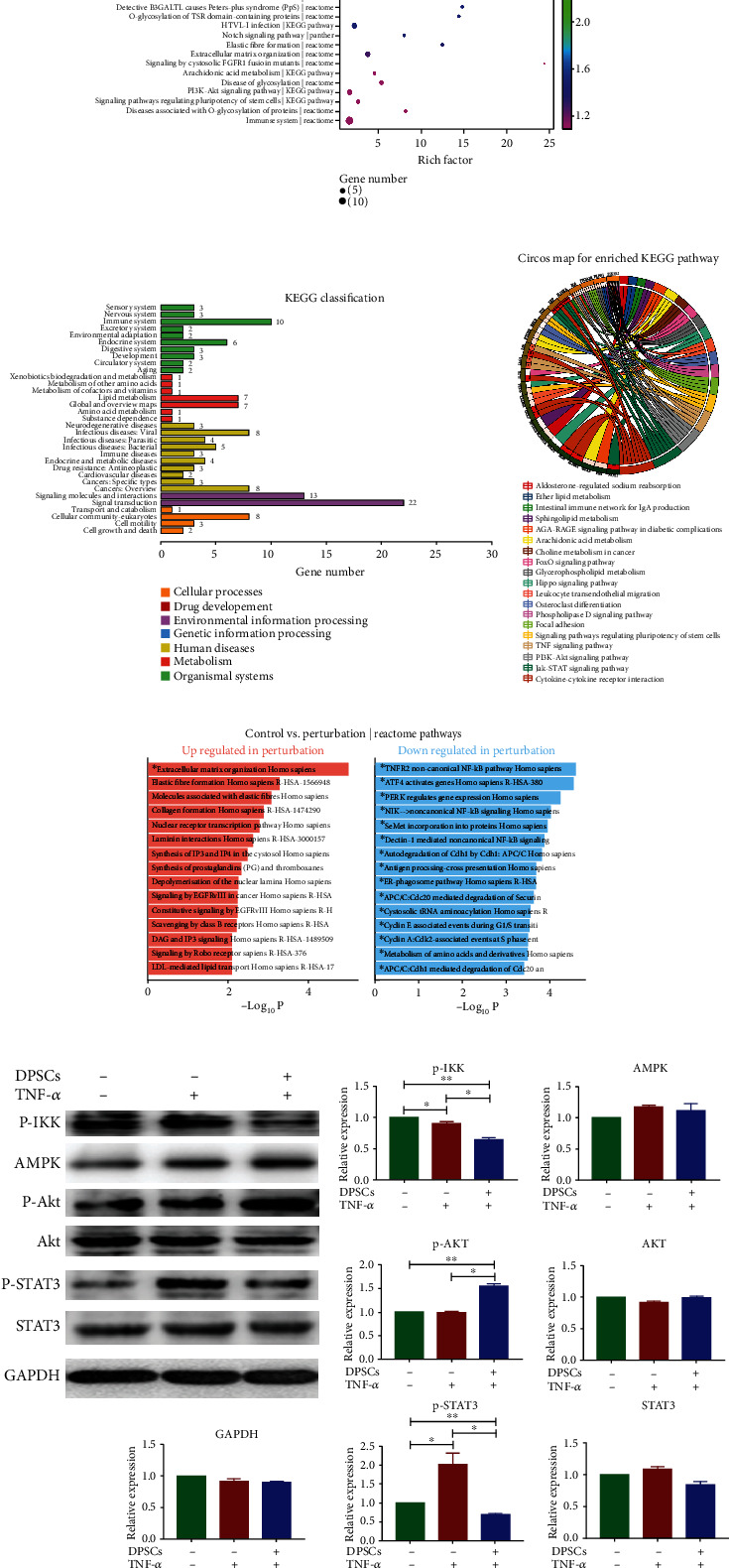
Pathway alteration after DPSC supernatant treatment. (a) Bubble diagram of the pathways enriched in the KEGG pathway, Reactome, and PANTHER databases. (b) KEGG classification of the DEGs after DPSC treatment. (c) Circos map of enriched KEGG pathways. (d) The interactive bar charts display the biological pathways that are overrepresented in the upregulated and downregulated genes generated by Enrichr. (e) Western blot results and gray value analysis results showing the pathway-related proteins in NPs after specific treatments. “TNF-*α*” represents NP cells cultured in 500 ng/ml TNF-*α*. “DPSC” represents NP cells cultured in culture media containing DPSC culture supernatant (1 : 3 v/v). After 2 days of treatment, cell lysates were analyzed by western blotting. Gray values were analyzed by ImageJ. “Relative expression” represents the gray value of a specific protein divided by that of GAPDH. ^∗^*p* < 0.05 and ^∗∗^*p* < 0.01. KEGG: Kyoto Encyclopedia of Genes and Genomes.

The results of the pathway enrichment analysis, based on the KEGG pathway, Reactome, and PANTHER databases, are presented in Figure 3(a). The top two terms with the highest statistical significance were “Jak-STAT signaling pathway” (KEGG pathway, 7/158, input genes/background genes, corrected *p* value =0.001773) and “signaling by interleukins” (Reactome, 10/400, input genes/background genes, corrected *p* value =0.001773).

Next, we took a closer look at the KEGG pathway results. The KEGG classification results for the DEGs are shown in Figure 3(b). Signal transduction, signaling molecules and interaction, lipid metabolism, and immune system alteration were obviously changed in the KEGG classification graph. In addition, a Circos graph showing the KEGG enrichment results is presented in Figure 3(c). The top three pathways enriched according to the KEGG enrichment analysis were the Jak-STAT signaling pathway (sea green lines, 7/158, input genes/background genes, corrected *p*-value =0.001773), cytokine-cytokine receptor interaction (chocolate lines, 8/263, input genes/background genes, corrected *p* value =0.002170), and TNF signaling pathway (burlywood lines, 5/110, input genes/background genes, corrected *p* value =0.011168).

Interestingly, the upregulated genes in the DPSCs' supernatant-treated group were enriched in the pathways of “extracellular matrix organization,” “elastic fiber formation,” and “collagen formation” (Figure 3(d), left). This result indicated that NP cells recovered after treatment. In addition, the downregulated genes were involved in NF-*κ*B activation pathways (such as the “TNFR2 noncanonical NF-*κ*B pathway,” “NIK noncanonical NF-*κ*B signaling pathway,” and “Dectin-1-mediated noncanonical NF-*κ*B signaling pathway”) (Figure 3(d), right). This result indicated that the inflammatory-related NF-*κ*B signaling pathway was downregulated after DPSCs treatment.

To determine the alterations caused by DPSCs treatment, western blotting was carried out. p-IKK and p-STAT3 were downregulated after DPSCs' supernatant treatment, indicating that the NF-*κ*B and JAK-STAT3 signaling pathways were inhibited (Figure 3(e)). The inhibition of these two prominent pathways verified the results of the DEG-enriched pathway analysis (Figure 3(d)). In addition, the Akt pathway was slightly increased, while AMPK remained unchanged.

### 3.5. DPSCs Treatment Restores NP Cells Function and Survival

**Figure 4:**
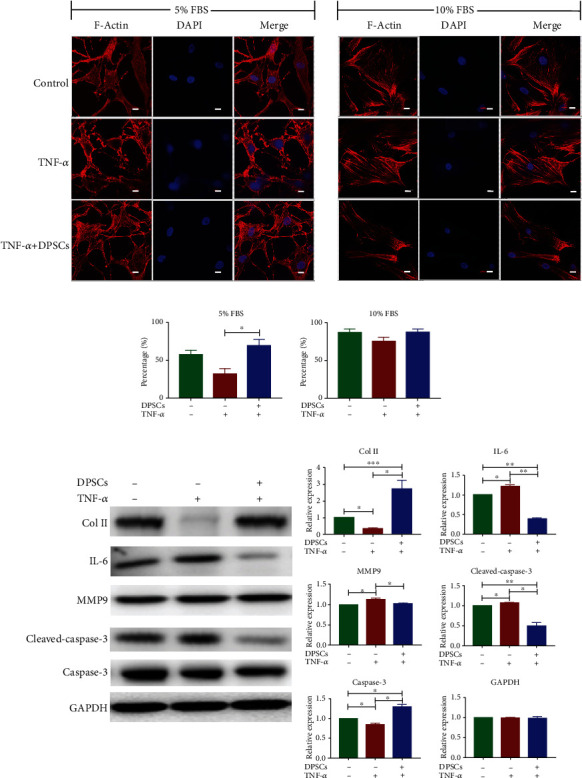
Biological effect of DPSCs supernatant on NP cells. (a) Confocal microscopy images of the cytoskeletal component (F-actin) distribution. White bar = 10 *μ*m. “TNF-*α*” represents NP cells cultured in 500 ng/ml TNF-*α*. “DPSCs” represent NP cells cultured in culture media containing DPSC culture supernatant (1 : 3 v/v). “5% FBS” indicates that NP cells were treated in culture media containing 5% FBS. “10% FBS” indicates that NP cells were treated in culture media containing 10% FBS. “Relative expression” represents the gray value of a specific protein divided by that of GAPDH. (b) Analysis of NP cells' cytoskeleton integrity. We calculated the percentage of cells with intact cytoskeleton/total cells counts. (c) Western blot results and gray value analysis results showing the proteins in NPs after specific treatments. To induce apoptosis, cells were treated in culture media containing 5% FBS. After 2 days of treatment, cell lysates were analyzed by western blotting. Gray values were analyzed by ImageJ. ^∗^*p* < 0.05, ^∗∗^*p* < 0.01, and ^∗∗∗^*p* < 0.001. FBS: fetal bovine serum; DAPI: 4,6-diamidino-2-phenylindole.

To mimic the environment of degenerated discs, NP cells were treated with 5% FBS (malnutrition status) with TNF-a (inflammatory status). Under such conditions, more NP cells appear to have disordered cytoskeleton and aggregated F-actin, indicators for strengthened apoptosis (Figures 4(a) and 4(b)). Cellular level of cleaved caspase-3 was also elevated (Figure 4(c)). However, DPSCs' supernatant treatment reduced apoptosis and partially stabilized the NP cell structure (Figure 4(b)). In addition, western blot analysis showed that DPSCs treatment increased the expression of collagen II and rescued TNF-*α*-induced IL-6 production (Figure 4(c)), suggesting functional recovery of NP cells after DPSCs' supernatant treatment. However, MMP9 was not evidently changed after treatment.

### 3.6. DPSCs Have Therapeutic Effects in the IVD Degeneration Rat Model

**Figure 5:**
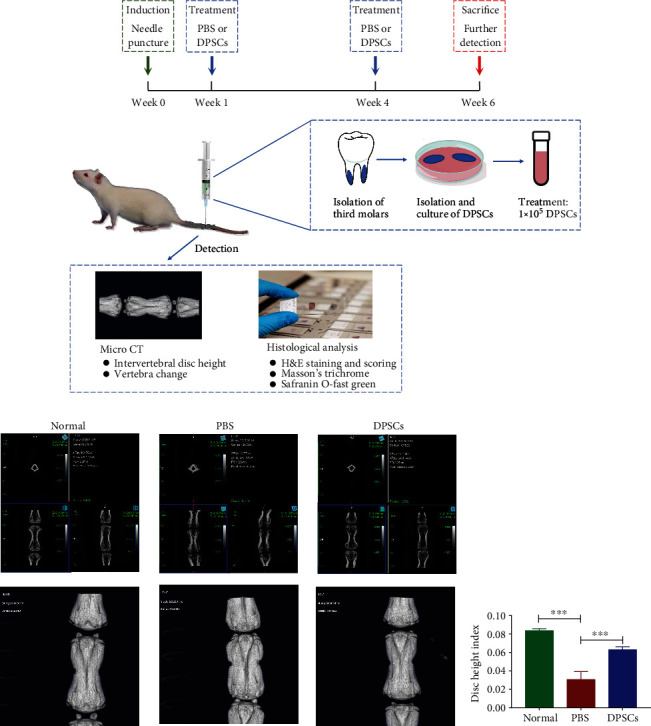
DPSC-based treatment scheme for treating IVD degeneration rats. (a) Flow diagram showing the design of the animal experiments. On week 0, a 20G needle was used to pierce the Co7-8 or Co8-9 intervertebral disc through the center of the disc until the opposite side. One week after the initial operation, 2 *μ*l of drugs (PBS or 5 × 10^4^ cell/*μ*l DPSCs) were injected into the intervertebral disc. The injection procedure was repeated at week 4. At week 6, radiological and histological analyses were carried out to evaluate the structural and architectural changes. (b) Typical micro-CT and 3D reconstruction images after DPSC treatment. The top pictures show the sagittal view, coronal view, and transverse view of the tail disc. The bottom images show the shrinking intervertebral disc height after different treatments. (c) Statistical graph of the DHI. ^∗∗∗^*p* < 0.001. DHI: disc height index.

The schematic diagram shown in Figure 5(a) depicts the time line and study design of the animal experiments. After establishment of the IVD model at week 0, PBS or 1 × 10^5^ DPSCs were administered to damaged IVDs at week 1 and week 4. When rats were sacrificed at week 6, vertebra samples were collected. The radiological and pathological changes caused by disc degeneration were then measured by micro-CT or staining.

As the micro-CT original image and 3D reconstruction demonstrated (Figure 5(b)), PBS-treated mice showed an obvious decrease in disc height between vertebrae and destruction of the end plate. It was also shown that DPSCs treatment could alleviate IVD degeneration. Since loss of disc height is used as a surrogate predictor of disc degeneration, we calculated the DHI (Figure 5(c)). The DHI was significantly decreased in the PBS treatment group compared with the normal group and was significantly increased after DPSCs treatment.

**Figure 6:**
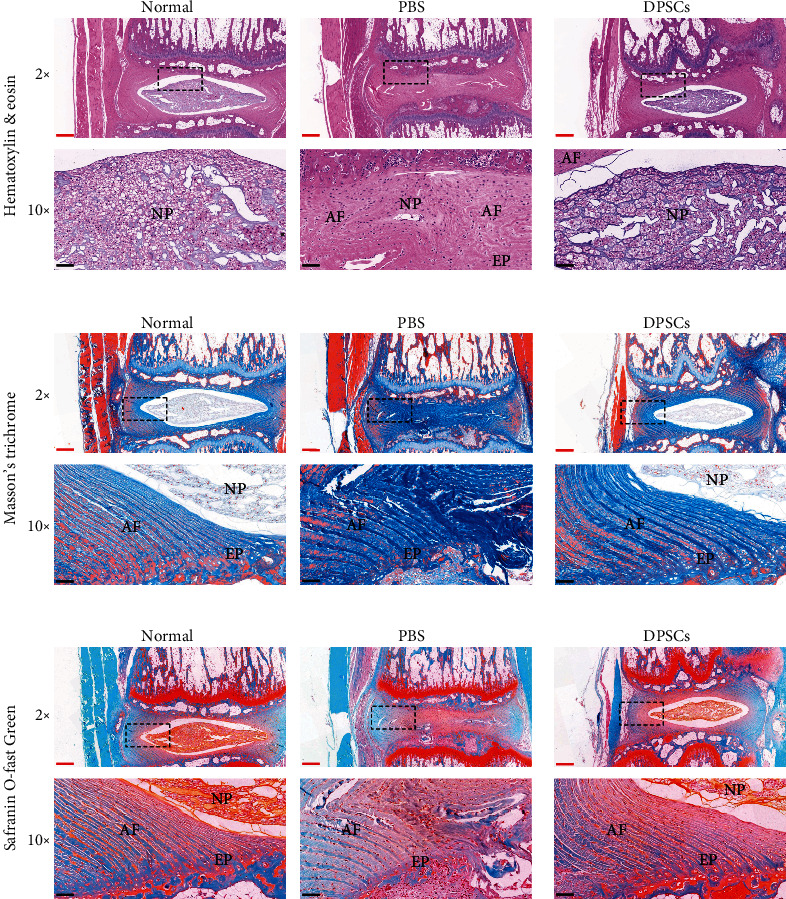
Histological evaluation of IVD degeneration rats. (a) H&E staining of the intervertebral disc at week 6. (b) Masson's trichrome staining of the intervertebral disc at week 6. (c) Safranin O-Fast Green staining of the intervertebral disc at week 6. The top images were photographed under 2× magnification. The bottom images are from the black dotted bordered rectangle under 10× magnification. Red bar = 500 *μ*m. Black bar = 100 *μ*m. AF: annulus fibrosus; NP: nucleus pulposus; EP: end plate.

Histological sections stained with hematoxylin and eosin (H&E) were used to observe the structures of the NP and AF, margin between the AF and NP, and cellularity and matrix of the NP (Figure 6(a)). In the PBS group, severe condensation of the normal gelatinous NP structure and serpentine AF structure could be observed. In addition, the numbers of NP cells and vacuoles were severely decreased in the PBS group with a very vaguely defined border between the NP and AF. These results indicated the successful induction of IVD degeneration and the lack of effect of the PBS injection. In contrast, the DPSCs treatment group showed improved NP and AF structures along with a clear border between the AF and NP. Masson's trichrome staining allowed detection of the changes in collagen fibers that could clearly depict the AF structure. The AF in the PBS group showed a ruptured structure and invasion to the endplate (Figure 6(b)). Safranin O staining showed less proteoglycan content and a more severe endplate distribution in the PSB group, which could be alleviated by DPSCs treatment (Figure 6(c)).

**Figure 7:**
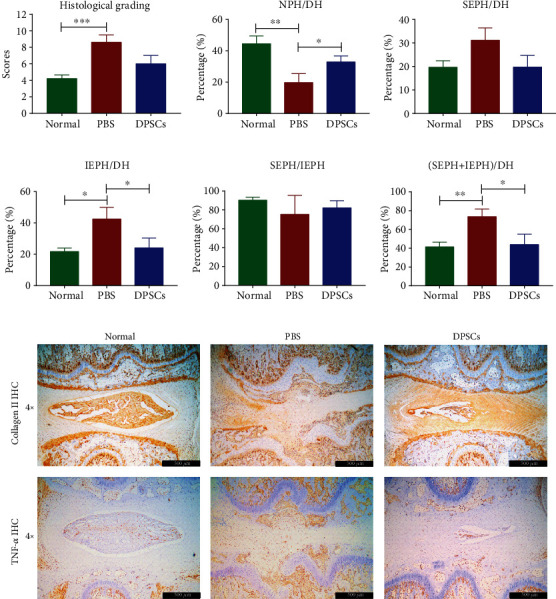
Structural analysis and immunobiological staining of IVD degeneration rats. (a) Histological scores representing the structural changes of the affected disc. The histological grading scale is the sum of the following 4 aspects: annulus fibrosus, border between the annulus fibrosus and nucleus pulposus, cellularity of the nucleus pulposus, and matrix of the nucleus pulposus, with each aspect ranging from 1 to 3. (b–f) Statistical analysis of the architectural parameters of the IVD. (b) NPH/DH reflects changes of the NP thickness. (c) SEPH/DH reflects changes of the superior endplate thickness. (d) IEPH/DH reflects changes of the inferior endplate thickness. (e) SEPH/IEPH reflects the changing ratio of the superior/inferior endplate thickness. (f) (SEPH+IEPH)/DH reflects alterations of the overall endplate thickness in one disc. (g) Immunohistological staining of TNF-*α* and collagen II in the intervertebral disc. Black bar = 500 *μ*m.^∗^*p* < 0.05, ^∗∗^*p* < 0.01, and ^∗∗∗^*p* < 0.001. NPH: maximum nucleus pulposus height; DH: intervertebral disc height; SEPH: superior endplate height; IEPH: inferior endplate height.

Quantitative analysis was carried out to evaluate the degeneration of each group. As Figure 7(a) shows, the histological score of the PBS group was significantly higher than those of the normal group and DPSCs treatment group. Regarding the IVD architecture (Figures 7(b)–7(f)), the IVDs in the PBS group had a significantly lower NPH/DH ratio and significantly higher (SEPH+IPEH)/DH and IPEH/DH ratios than the IVDs in the DPSCs treatment group. However, the changes in the SEPH/DH and SEPH/IEPH ratios were not significant between the groups. These results indicate shrinkage of the NP and an abnormal thickness of the IEPH after needle piercing, which could be alleviated by DPSCs treatment.

Immunohistological staining of TNF-*α* demonstrated that more TNF-*α* were released in the PBS group compared with the normal group. DPSCs treatment could reduce the production of TNF-*α* which correlated with the previous results. Besides, more ECM (Col II) was produced after DPSCs treatment compared with the PBS group (Figure 7(g)).

## 4. Discussion

Increased levels of proinflammatory cytokines, such as TNF-*α*, IL-1*β,* and IL-6, are an important characteristic and pathogenic factor for IVD degeneration [27]. These proinflammatory molecules, which can be secreted by NP cells, AF cells, macrophages, and T cells, can trigger a range of pathogenic responses, including autophagy, senescence, or apoptosis [6, 28]. These cytokines can also induce catabolic activity with enhanced expression of matrix-degrading enzymes and degradation of collagen and aggrecan, leading to IVD structural damage [29]. In our experiment, we found that the establishment of inflammatory status by TNF-*α* could promote the induction of IL-6 and apoptosis (together with low nutrition), which are harmful to NP cell survival and function.

As previously described, TNF-*α* has been reported to be strongly linked to the pathogenesis of IVD degeneration [30, 31]. TNF-*α* can bind to TNF receptor 1, permitting the recruitment of an adaptor protein, TNFR1-associated death domain protein (TRADD), which serves as a docking site for TNF-receptor-associated factor 2 (TRAF2) and receptor-interacting serine/threonine protein kinase 1 (RIP1). This process can activate the downstream NF-*κ*B signaling pathway, which plays a crucial role in mediating the production of other proinflammatory cytokines, such as IL-1*β* and IL-6. Interestingly, our results showed that DPSCs' supernatant treatment could reverse the activation of the TNF-induced NF-*κ*B pathway. Deactivation of NF-*κ*B reduced the production of proinflammatory cytokines (such as IL-6) and chemokines (such as CCL-7) and impaired the activation of the JAK-STAT signaling pathway. The IHC results showed that local production od TNF-*α* was decreased after DPSCs treatment, which could restore local immune balance and stabilize inflammatory status.

The binding of TNF-*α* to its receptor can also lead to the formation of death-inducing signaling complex (DISC), activating Fas-associating protein, which has a novel death domain (FADD), and downstream cysteinyl aspartate-specific proteinase-3 (Caspase-3), inducing apoptosis of NP cells [27, 32]. In our experiment, we found that DPSCs' supernatant could reduce TNF-*α*-induced NP cell apoptosis and cleavage of the substrate actin and maintain cell shape. Hence, DPSCs treatment protects NP cell survival under inflammatory conditions.

In addition, TNF-*α* inhibits the expression of type II collagen-encoding genes [33], which play a critical role in the maintenance of the NP tissue matrix. In our experiment, we found that DPSCs treatment could promote the production of type II collagen and help to maintain a relatively intact structure of the IVD.

As previously reported, DPSCs possess stronger capacities for immunomodulation and self-renewal than BMSCs [34, 35]. In terms of their strong immune modulating capacity, DPSCs have previously been reported to be capable of suppressing T-cell proliferation and enhancing anti-inflammatory M2 macrophage phenotype polarization [36, 37]. These characteristics of DPSCs are therapeutically effective in many kinds of immune-related diseases, such as rheumatoid arthritis [38], systemic lupus erythematosus [39], and periodontitis [16]. However, whether DPSCs can be used to treat IVD degeneration remains to be elucidated.

DPSCs have been reported to have therapeutic potential in treating rheumatoid arthritis, periodontal disease, ischemic stroke, etc. [16, 38, 40]. In our study, we found that DPSCs may be beneficial in treating IVD degeneration. DPSCs treatment had a therapeutic effect and improved the NP and AF structures, alleviated extracellular matrix degradation and endplate distribution, and increased collagen fiber expression. Regarding the IVD architecture, DPSCs could protect the integrity of the IVD structure and mitigate NP shrinkage and abnormal architecture. In vitro experiments showed that DPSCs could deactivate the inflammation-related NF-*κ*B and JAK-STAT pathways and reduce IL-6 production, which helped to control local inflammatory status. Moreover, NP cell function was also restored with elevated expression of collagen II and reduced levels of apoptosis.

Although both reduction of cell apoptosis and inflammation play roles in the protective effect of DPSCs' supernatant, yet according to gene sequencing and in vitro results, we believe that regulation of inflammation is of higher priority. Furthermore, knocking down MALAT1, a kind of lncRNA which has been reported to have immunosuppressive properties, could attenuate the immune-suppressive effect of DPSCs which also indicated that the immune regulation effect of DPSCs is more important in our study (data not shown).

Therefore, in this study, we described the therapeutic effect of DPSCs in an IVD degeneration rat model, proposed the underlying molecular mechanism of DPSC treatment, and provided a novel cell therapy involving DPSCs injection for IVD degeneration treatment. However, since the degeneration rat model may not fully mimic the pathogenesis in humans, further clinical research needs to be carried out to verify the above conclusions.

## 5. Conclusion

In this study, we proposed the underlying molecular mechanism of DPSCs treatment, verified the therapeutic effect of DPSCs in an IVD degeneration rat model, and provided a novel cell therapy involving DPSCs injection for IVD degeneration treatment.

## Data Availability

All data generated and/or analyzed during this study are included in this published article. Data sharing is not applicable to this article as no datasets were generated or analyzed during the current study. However, the data that support the findings of this study are available from the corresponding author upon reasonable request.

## Supplementary Materials

Supplementary 1 Supplementary Figure 1: the identification of DPSCs' markers and differentiation ability. (A) Representative image of DPSCs. (B) Adipogenesis ability of DPSCs after induction. (C) Osteogenesis ability of DPSCs after induction. (D) Surface marker of DPSCs.

Supplementary 2 Supplementary Figure 2: GO annotation of the DEGs from control NP cells and NP cells after DPSC supernatant treatment. (A) Differential gene GO function classification map after DPSC treatment. (B) GO hierarchy map of the terms enriched in “molecular function.”

Supplementary 3 Supplementary Table 1: since the primer/adaptor contaminated reads, low-quality reads, and N overtop reads were filtered out, the clean reads had better quality than the individual forward or reverse reads. Supplementary Table 1 showed the summary for data filtering.

Supplementary 4 Supplementary Table 2: with respect to alignment, the average percentage of paired reads was 97.87% (all data >70%), and the average percentage of multiple paired reads was 3.21% (all data <10%), which met the quality criteria for subsequent analysis. Supplementary Table 2 showed the number of clean reads per sample and percentage alignment to hisat2.
