# Supplementary figures and images for: DPSCs Protect Architectural Integrity and Alleviate Intervertebral Disc Degeneration by Regulating Nucleus Pulposus Immune Status
Source: Stem Cells Int. 2022 Oct 15;2022:7590337. doi: 10.1155/2022/7590337 (PMC9590116; doi:10.1155/2022/7590337)

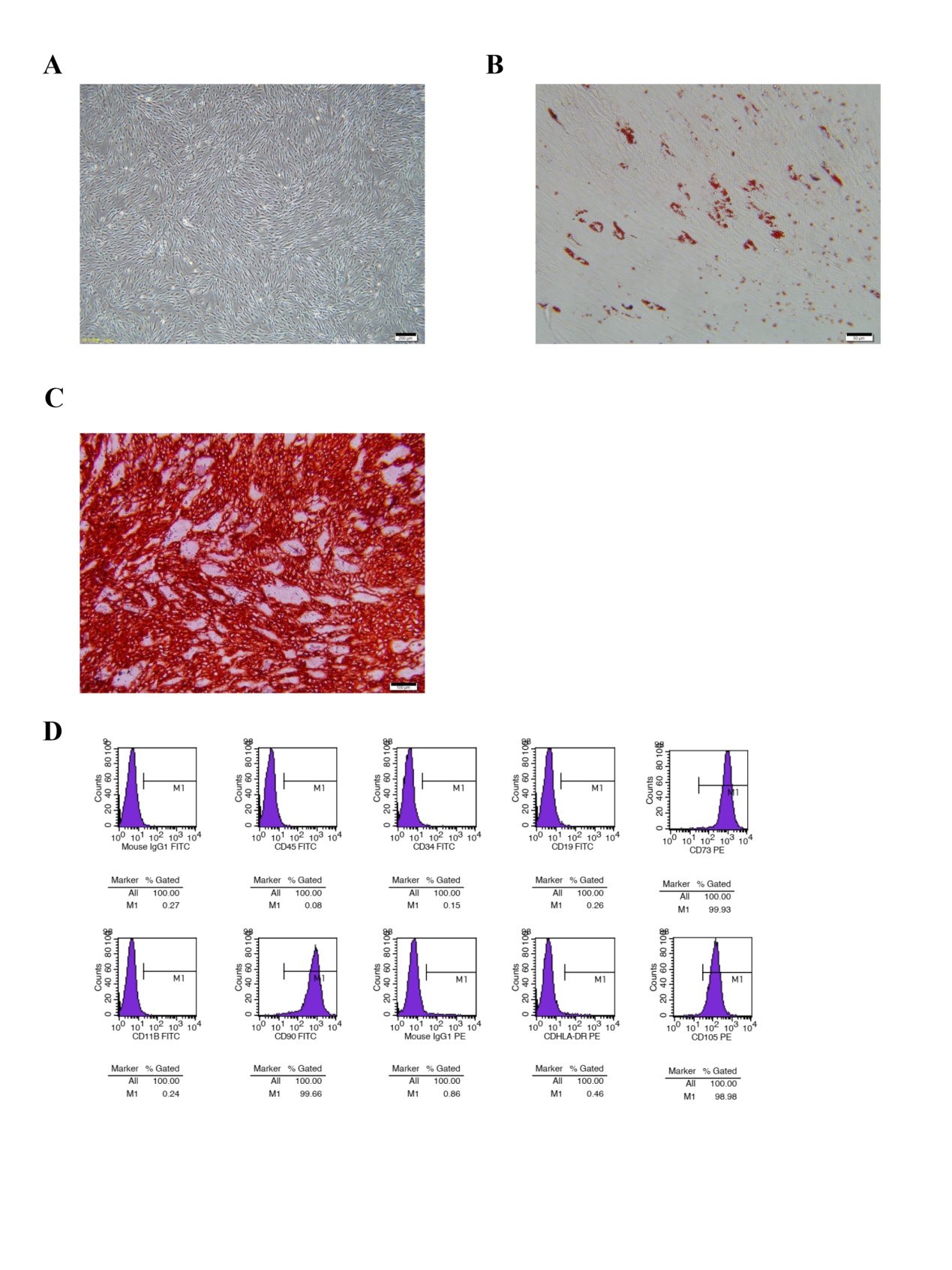

Supplement: Supplementary 1 — Supplementary Figure 1: the identification of DPSCs' markers and differentiation ability. (A) Representative image of DPSCs. (B) Adipogenesis ability of DPSCs after induction. (C) Osteogenesis ability of DPSCs after induction. (D) Surface marker of DPSCs. [file 7590337.f1.docx]

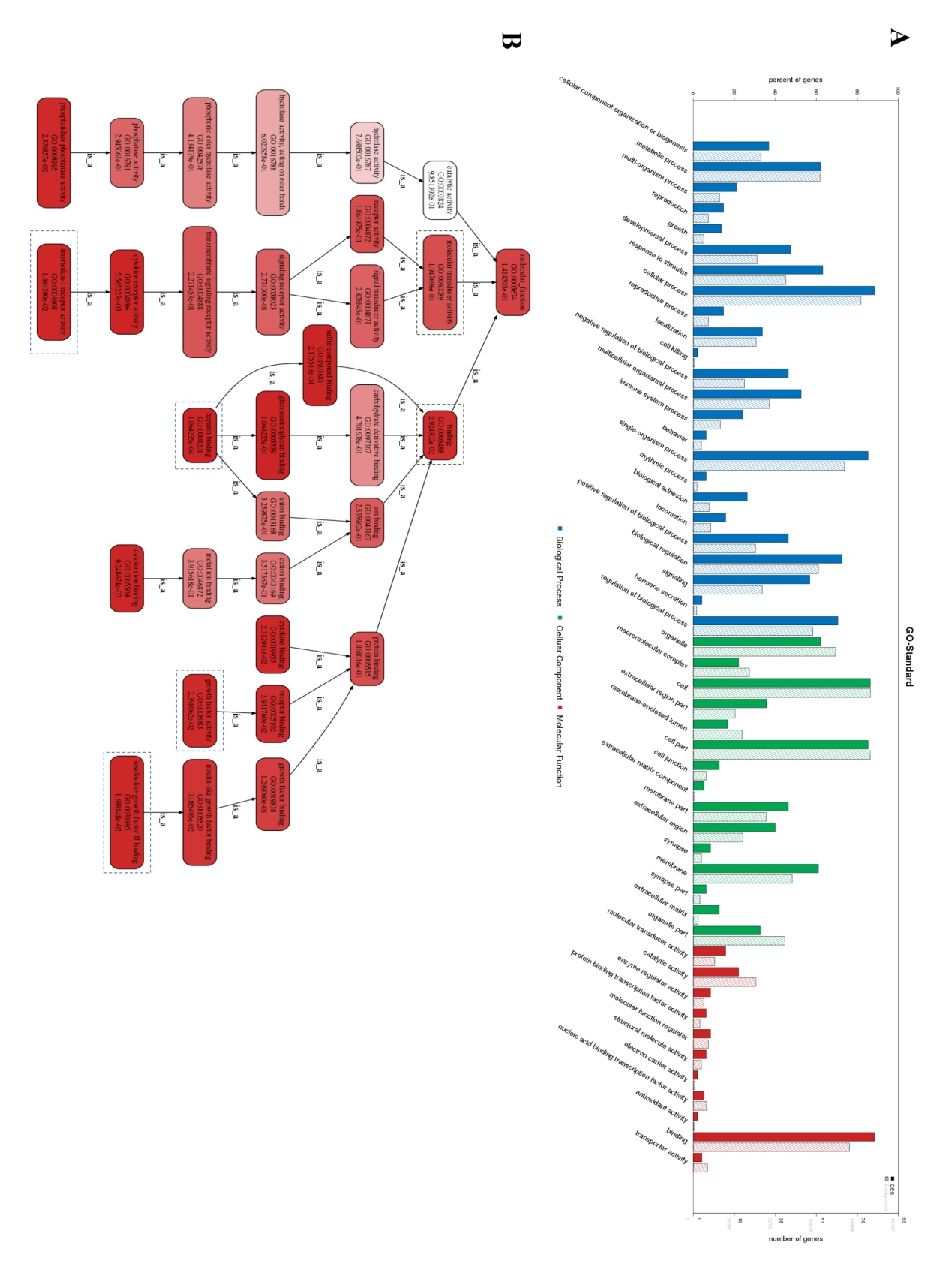

Supplement: Supplementary 2 — Supplementary Figure 2: GO annotation of the DEGs from control NP cells and NP cells after DPSC supernatant treatment. (A) Differential gene GO function classification map after DPSC treatment. (B) GO hierarchy map of the terms enriched in “molecular function.” [file 7590337.f2.docx]
