# Supplementary material for: DPSCs Protect Architectural Integrity and Alleviate Intervertebral Disc Degeneration by Regulating Nucleus Pulposus Immune Status
Source: Stem Cells Int. 2022 Oct 15;2022:7590337. doi: 10.1155/2022/7590337 (PMC9590116; doi:10.1155/2022/7590337)
Supplement: Supplementary 3 — Supplementary Table 1: since the primer/adaptor contaminated reads, low-quality reads, and N overtop reads were filtered out, the clean reads had better quality than the individual forward or reverse reads. Supplementary Table 1 showed the summary for data filtering. [file 7590337.f3.docx]

**Supplementary Table 1 Summary for data filtering**

| **Sample** | **Raw reads** | **Clean reads** | **Clean rate (%)** | **Q20 (%)** | **Q30 (%)** |
| --- | --- | --- | --- | --- | --- |
| Crtl 1 | 49064554 | 47983552 | 97.57 | 98.42 | 95.02 |
| Crtl 2 | 48816026 | 46548718 | 95.13 | 98.52 | 95.41 |
| Crtl 3 | 48303664 | 45498252 | 93.33 | 98.59 | 95.52 |
| DPSCs 1 | 48159084 | 47144586 | 97.69 | 98.14 | 94.13 |
| DPSCs 2 | 45597072 | 43817258 | 95.73 | 98.58 | 95.52 |
| DPSCs 3 | 46664378 | 45277900 | 96.39 | 98.67 | 95.65 |

Q30 indicates that the sequencing error rate of this base is 0.1%.

Q30(%): In Clean reads, the percentage of bases with a quality value greater than Q30

Q20 indicates that the sequencing error rate of the base is 1%

Q20(%): In Clean reads, the percentage of bases with a quality value greater than Q20
