# Supplementary material for: DPSCs Protect Architectural Integrity and Alleviate Intervertebral Disc Degeneration by Regulating Nucleus Pulposus Immune Status
Source: Stem Cells Int. 2022 Oct 15;2022:7590337. doi: 10.1155/2022/7590337 (PMC9590116; doi:10.1155/2022/7590337)
Supplement: Supplementary 4 — Supplementary Table 2: with respect to alignment, the average percentage of paired reads was 97.87% (all data >70%), and the average percentage of multiple paired reads was 3.21% (all data <10%), which met the quality criteria for subsequent analysis. Supplementary Table 2 showed the number of clean reads per sample and percentage alignment to hisat2. [file 7590337.f4.docx]

Supplementary Table 2 Number of clean reads per sample and percentage alignment to hisat2

| **Samples** | **Clean reads** | **Aligned reads** | **Uniquely aligned reads** | **Multiple aligned reads** |
| --- | --- | --- | --- | --- |
| Crtl 1 | 47983552 (100.00%) | 46968858 (97.89%) | 45496196 (96.86%) | 1472662 (3.14%) |
| Crtl 2 | 46548718 (100.00%) | 45242983 (97.19%) | 43507789 (96.16%) | 1735194 (3.84%) |
| Crtl 3 | 45498252 (100.00%) | 44631098 (98.09%) | 43242133 (96.89%) | 1388965 (3.11%) |
| DPSCs 1 | 47144586 (100.00%) | 46280605 (98.17%) | 44982518 (97.20%) | 1298087 (2.80%) |
| DPSCs 2 | 43817258 (100.00%) | 42736932 (97.53%) | 41208263 (96.42%) | 1528669 (3.58%) |
| DPSCs 3 | 45277900 (100.00%) | 44540302 (98.37%) | 43302744 (97.22%) | 1237558 (2.78%) |
